# Identification and characterization of new B cell epitopes on the nucleocapsid protein of bovine coronavirus using monoclonal antibodies

**DOI:** 10.3389/fcimb.2026.1881944

**Published:** 2026-07-24

**Authors:** Linlin Fang, Shuang Liu, Xiaohua Wang, Feng Zhang, Jiarong Yu, Xiaohan Zhang, Yaqin Dong, Jingyue Bao

**Affiliations:** China Animal Health and Epidemiology Center, Qingdao, China

**Keywords:** B cell epitopes, BCoV, diagnostic target, monoclonal antibodies, N protein

## Abstract

Bovine coronavirus (BCoV) is an important pathogen that causes diarrhea in calves, winter dysentery in adult cattle and respiratory diseases, causing significant economic losses to the global cattle industry. N protein is the most conserved structural protein of coronavirus, with strong immunogenicity and serving as an ideal target for early diagnosis and vaccine development. In this study, the prokaryotic expression system was used to express the recombinant BCoV N protein and used it as an immunogen to immunize BALB/c mice. Two specific monoclonal antibodies (mAbs) against BCoV N protein, 4D8 and 6F3, were successfully generated through hybridoma technology. Western blotting and IFA results demonstrated that both mAbs can react specifically with BCoV and have good reactivity and specificity. Isotype identification revealed that both monoclonal antibodies belong to the IgG1 isotype. By constructing a series of truncated N protein fragments and subsequent western blot analysis, the linear B cell epitopes recognized by these mAbs were precisely mapped. The research results showed that the mAb 4D8 can identify the epitope spanning ^157^TPADILD^163^, while the mAb 6F3 recognized the epitope spanning ^378^NAYQQQDGMM^387^. Homology analysis shows that these two epitopes are highly conserved in different BCoV reference strains. Structural positioning analysis using SWISS-MODEL and PyMOL software shows that both epitopes were exposed on the surface of BCoV N protein. This study successfully generated two specific mAbs and identified two new linear B cell epitopes, which provided a valuable tool for further investigation of the structure and function of BCoV N protein, and establish an important foundation for the development of specific epitope-based BCoV diagnostic methods.

## Introduction

Bovine coronavirus (BCoV) is an important pathogen that causes diarrhea and respiratory diseases in cattle (van den Hurk et al., 2024). Depending on the viral strain and the site of infection, BCoV can cause three major disease syndromes: calf diarrhea, winter dysentery in adult cattle and bovine respiratory disease complex (Vlasova and Saif, 2021; Saif and Jung, 2020). BCoV infection not only affects the growth and development of calves, but also leads to a decreased milk production in cows and reduced weight gain in beef cattle (Saif, 2010; Saif and Jung, 2020). BCoV is distributed globally, causing serious economic losses to farms and affecting the sustainable development of livestock industry (Zhu et al., 2022; Bartels et al., 2010; Burimuah et al., 2020; Gagea et al., 2006; Kanno et al., 2007; Keha et al., 2019). Although the mortality rate of BCoV infection is low, the morbidity rate is generally high, and the epidemic mainly occurs in autumn and winter (Lojkić et al., 2015; Park et al., 2006; Decaro et al., 2008). Statistical analysis indicate that the detection rate of BCoV in calf feces is relatively high, ranging from 0% to 46% in healthy calves and from 3.4% to 69.0% in calves with diarrhea (Zhu et al., 2022).

The BCoV genome encodes five major structural proteins: spike (S) protein, hemagglutinin-esterase (HE), membrane (M) protein, envelope (E) protein, and nucleocapsid (N) protein (Lai and Cavanagh, 1997). N protein is encoded by a 1, 347 bp gene and contains 448 amino acids. N protein is the most expressed viral antigen in coronavirus-infected cells (Hurst et al., 2013). N protein plays a key regulatory role in the replication and assembly of viruses (Surjit et al., 2006). Its main function is to encapsidate the viral genome into a shell-like structure to protect and stabilize genetic material. In addition, N protein interacts with various viral components (Narayanan et al., 2003) and host factors (He et al., 2004; Kopecky-Bromberg et al., 2007), to enhance the infectivity and pathogenicity of the virus. Studies show that N protein can be expressed efficiently in the prokaryotic system and has strong immunogenicity, which is an ideal target for the development of BCoV diagnostic kits (Narayanan et al., 2003).

In this study, the N protein of BCoV was successfully and efficiently expressed using the pET-32a/BL21 system. Using recombinant BCoV N protein as an antigen, two monoclonal antibodies(mAbs) against N protein were successfully generated. The results of immunofluorescence assay (IFA) and western blotting demonstrated that both mAbs have good specificity. Antigen epitope analysis identified two distinct novel antigen epitopes. The generation of these mAbs facilitates in-depth investigation of the immunological and structural characteristics of N proteins, and lays the foundation for the development of BCoV immunodiagnosis technology.

## Materials & methods

### Viruses and cells

BCoV strain NM01 was isolated and identified in our laboratory from a diarrheic calf fecal sample in Inner Mongolia, China, in 2021. The strain has been maintained in our laboratory, and its identity was confirmed by RT-PCR targeting the N gene and sequencing analysis. Human cecoileal colorectal carcinoma cells (HCT-8) were cultured in Roswell Park Memorial Institute 1640 Medium (RPMI 1640, Biological Industries) containing 10% fetal bovine serum (FBS, Vazyme) at 37°C in a humidified atmosphere containing 5% CO_2_. To propagate BCoV, HCT-8 cells were cultured in RPMI 1640 supplemented with 8 μg/mL trypsin. Mouse myeloma cell line SP2/0 was preserved at the China Animal Health and Epidemiology Institute (Qingdao, China).

### Preparation of immunogen

HCT-8 cells were cultured in cell culture flasks using RPMI 1640 medium containing 10% FBS. When the cells reached to 90%, inoculated the BCoV virus. After 36 h, the virus supernatant was collected and centrifuge at 8, 000 rpm at 4°C for 10 minutes to remove cell fragments. After high-speed centrifugation, the supernatant was discarded, the pellet was collected and resuspended in PBS. The protein concentration was determined, and the samples were aliquoted and stored at −80 °C for future use.

### Prokaryotic expression of full-length and truncated overlapping fragments of N protein

In order to obtain recombinant BCoV N protein, the recombinant plasmid pET32a-N (BCoV China/BCoV cattle B298/2021, GenBank accession No. OP866728.1) was transformed into E. coli BL21 (DE3) (Sangon, Shanghai, China), and cultured in Luria-Bertani liquid culture medium containing 100 µg/mL ampicillin until the optical density of the culture reaches 0.5 at 600 nm (OD_600_). N protein expression was induced with 1 mM isopropyl-β-D-1-thiogalactopyranoside (IPTG) at 37 °C for 5 h, and then the detection was carried out by sodium dodecyl sulfate-polyacrylamide (SDS-PAGE) analysis using cell lysates. Recombinant N protein was purified using a Ni-NTA His-Bind Resin kit (Millipore, USA) under non-denaturing conditions.

### Construction of truncated N protein fragments

To map the epitopes recognized by anti-N mAbs, a series of truncated N protein fragments with His tags were constructed using pET-32a plasmids. The BCoV N protein gene was initially truncated into six overlapping fragments: N1 (aa 1-80), N2 (aa 76-160), N3 (aa 156-240), N4 (aa 226-320), N5 (aa 315-400), and N6 (aa 395-449). These fragments were expressed in E. coli BL21(DE3) cells. For further fine mapping, additional truncated fragments (N7-N17 for the N3 region and N19-N30 for the N5 region) were generated using the same strategy.

### Generation of monoclonal antibodies against BCoV N protein

Four-week-old female BALB/c mice were intraperitoneal injected 50 μL of recombinant BCoV N protein (2 mg/mL, equivalent to 100 μg per mouse) mixed with an equal amount of Freund’s complete adjuvant (Sigma, USA). Subsequently, the same dose of N protein mixed with Freund’s incomplete adjuvant was injected every 2 weeks, and co-immunized 3 times. Three days after the last immunization, the mice were euthanized by cervical dislocation under isoflurane anesthesia, the spleens were aseptically removed, and the splenocytes were isolated. Splenocytes and SP2/0 myeloma cells were fused using polyethylene glycol 4000 (PEG4000, Sigma, USA) at a ratio of 10:1. After fusion, the cells were resuspended in HAT selective medium (containing 100 μM hypoxanthine, 0.4 μM aminopterin, and 16 μM thymidine) and seeded into 96-well plates at a density of approximately 2 × 10^5^ cells per well. Hybridoma clones were screened by indirect ELISA using recombinant BCoV N protein (2 μg/mL) as the coating antigen. The culture supernatants from wells with visible hybridoma colonies were collected 10–14 days after fusion and tested for antibody reactivity. Positive wells were defined as those yielding an OD_450_ value at least 2.1-fold greater than the negative control (SP2/0 culture supernatant). The positive hybridoma cells were then subjected to limiting dilution cloning at a density of 0.5–1 cell per well in 96-well plates. After each round of subcloning, the clones were re-screened by ELISA to confirm stable antibody secretion. This cloning procedure was repeated three times to ensure monoclonality. Positive hybridoma cells were cloned and cultured three times to ensure monoclonality. Monoclonal hybridoma cells in the logarithmic growth period were frozen at a density of 1×10^6^/mL for later use. To generate mAbs, after amplifying the hybridoma cell line, the mouse ascites method was used to produce monoclonal antibodies. Monoclonal antibodies purified from ascites were characterized and identified by western blotting and indirect immunofluorescence assay (IFA).

### Enzyme-linked immunosorbent assay

Recombinant BCoV N protein was diluted to 2 μg/mL in carbonate buffer were used to coat 96-well plates overnight at 4°C. After each step, the plates were washed three times with PBST to remove unbound materials. The plates were blocked with 5% skimmed milk in PBST for 2 h at 37°C. After washing 3 times with PBST, 100 μL of two-fold dilutions of serum or hybridoma culture supernatant were added to each well as the primary antibody, respectively, and incubated at 37°C for 1h. Goat anti-mouse HRP-IgG 1:5000 dilution was used as the secondary antibody, incubated at 37°C for 50 min. TMB was used as the color development solution, incubated at 37 °C for 15min in the dark, and then the reaction was stopped by the addition of 1M H_2_SO_4_; the OD_450_ value was detected by using an ELISA plate reader (Tecan, Switzerland).

### Identification of monoclonal antibody subclasses

Antibody subclasses were determined using the SBA Clonotyping System-HRP Antibody Subclass Identification Kit, following the manufacturer’s instructions.

### Western blotting analysis

BCoV protein expression identification and the epitope mapping of mAbs were determined by Westernting blot analysis. The truncated N protein and BCoV sample were separated on a 12% SDS-polyacrylamide gel and then transferred to polyvinylidene difluoride (PVDF) membranes (Vazyme). To prevent non-specific binding, the membranes were sealed at room temperature with a PBST solution containing 5% skimming milk for 2 h. The monoclonal antibody prepared in this study was used as the primary antibody at 1:2000 for an overnight incubation at 4°C. After washing with PBST three times, the membranes were treated with HRP-labelled goat anti-mouse IgG diluted 1:2000 for 1h at room temperature. Proteins were detected using enhanced chemiluminescence (ECL) reagents (Vazyme). The images were acquired using a western blotting detection system (Bio-Rad, USA).

### Immunofluorescence assay

HCT-8 cells were plated in a 12-well plate and infected with BCoV at a multiplicity of infection (MOI) of 0.1. When the cytopathic effect (CPE) became evident, the cells were gently washed with PBS and fixed with 4% paraformaldehyde (Beyotime) for 20min at room temperature. The cells that had been fixed were permeabilized with 0.1% Triton X-100 (Beyotime) for 5 min at room temperature. After three washes with PBS, then mAbs (diluted at 1:200) were introduced to the cell plates and then incubated overnight at 4°C. After another three washes, the cells were incubated with Alexa Fluor 488-labeled Goat Anti-Mouse IgG (H+L) (Beyotime) for 50 min at 37°C in the dark. After another three washes, the cells were incubated with Antifade Mounting Medium (Beyotime) in the dark for 5 min at room temperature. Finally, use a fluorescence microscope (Leica, Germany) for observation.

### Epitope mapping of anti-BCoV N protein monoclonal antibodies

To determine the minimal binding regions of the mAbs, bacterial lysates containing various truncated N protein fragments were separated by SDS-PAGE and transferred onto PVDF membranes. The membranes were blocked with 5% skim milk in PBST and then incubated overnight at 4 °C with either mAb 4D8 or mAb 6F3 as the primary antibody. After washing, the membranes were incubated with HRP-conjugated goat anti-mouse IgG (H+L) for 1 hour at room temperature. Protein bands were visualized using an ECL substrate. Positive immunoreactivity with a specific truncated fragment helped narrow down the region containing the epitope. This process was repeated iteratively using progressively smaller overlapping fragments until the minimal linear epitope was identified.

### Sequence conservation analysis

To evaluate the conservation of the identified epitopes, multiple sequence alignment was performed using Jalview software (Version 2.11.2.6) (Waterhouse et al., 2009). The amino acid sequences of the identified epitopes were compared with the corresponding regions of BCoV N protein sequences from 20 reference strains available in GenBank, including isolates from China, USA, Canada, Japan, South Korea, Germany, Italy, and Belgium. The GenBank accession numbers and countries of origin for all reference strains are listed in **Table 1**. Sequence alignment was conducted using the ClustalW algorithm with default parameters.

**Table 1:** BCoV reference strains used for sequence conservation analysis of the N protein epitopes.

Strain name	GenBank accession No.	Country of origin
China/BCoV/cattle/B298/2021	OP866728.1	China
HLJ-14/CHN	KM985634.1	China
FRA/EPI/Caen/2004/14	KT318096.1	France
KCD/GJ1/2019	MZ822367.1	Korea
KCD/CW11/2021	OL840255.1	Korea
China/BCoV-cattle-F226/2021	OP866727.1	China
China/BCoV-cattle-B277a/2021	OP866729.1	China
BCoV/NMG1/2022	OP924545.1	China
FRA/EPI/Caen/2013/10	KT318092.1	France
BCoV-2014-13	KX982264.1	France

### Structural modeling and analysis of antigenic epitopes

To investigate the structural characteristics of the identified epitopes, the secondary structure, hydrophilicity, and surface accessibility of the BCoV N protein were predicted using DNAStar Protean 7.0 software. The tertiary structure of the BCoV N protein was predicted using the SWISS-MODEL homology modeling server (Waterhouse et al., 2018). A homology model was generated using the AlphaFold DB model of A0A7M4CG34 as the template (UniProtKB entry, organism: unknown), which shares 98.88% sequence identity with the BCoV N protein. The quality of the generated model was assessed using the GMQE (Global Model Quality Estimation) score provided by SWISS-MODEL, with the model achieving a GMQE score of 0.67. The positions of the two antigenic epitopes were mapped onto the predicted three-dimensional structural model using PyMOL software (Schrödinger, LLC), and their spatial distribution within the N protein was analyzed.

## Results

### Production of mAbs against BCoV N protein

**Figure 1:**
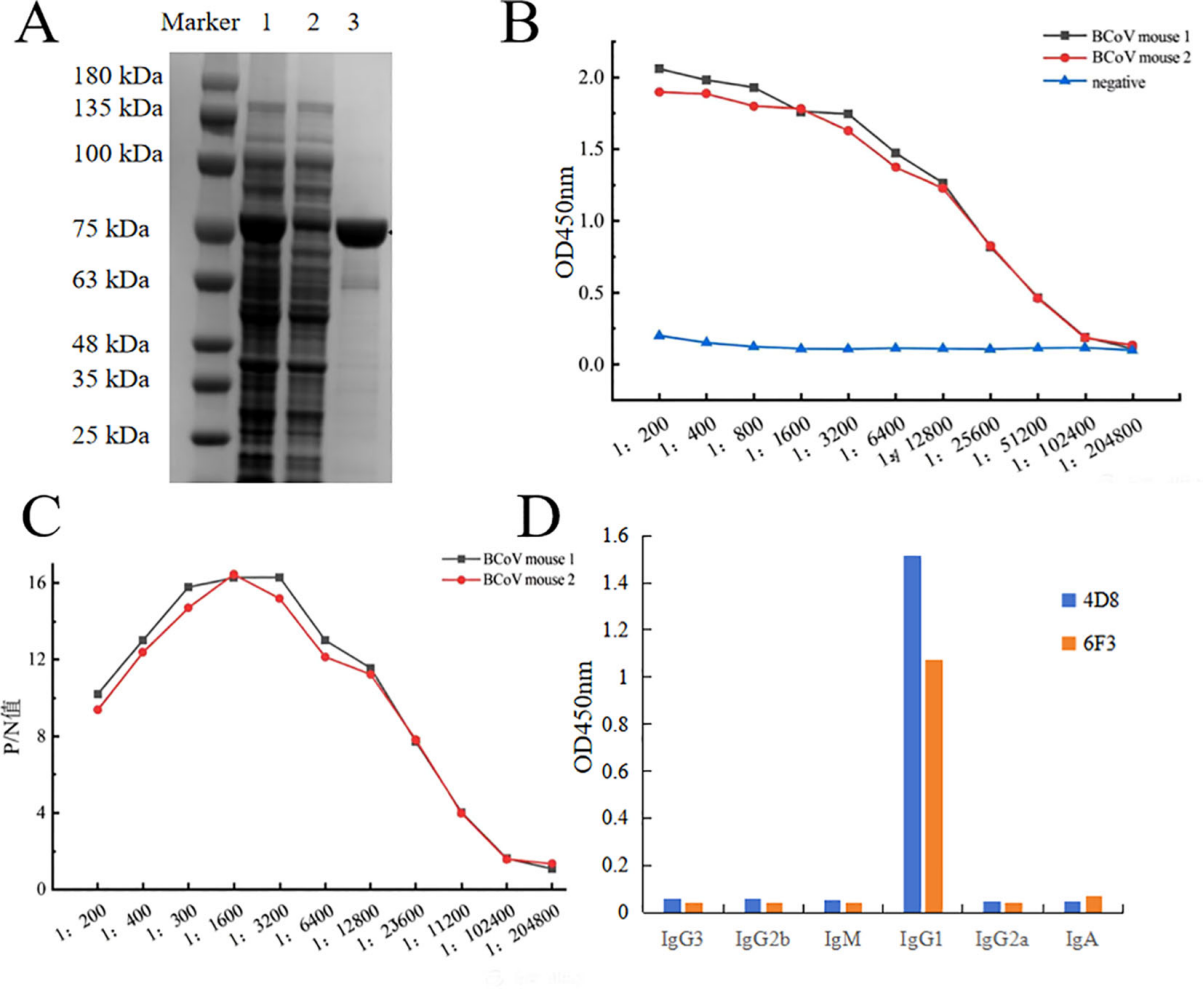
Generation of BCoV N protein-specific mAbs 4D8 and 6F3. **(A)** SDS-PAGE analysis of BCoV N protein purified by affinity chromatography. The gel was stained with Coomassie Brilliant Blue. Marker lane: 25 kDa-180 kDa marker protein bands; Lane 1: cell lysate showing a prominent band of the expressed protein of about 75 kDa; Lane 2: flow-through fraction; Lane 3: affinity purified full-length N protein of about 75 kDa. **(B, C)** Serum antibody titers against BCoV N protein from immunized mice were measured by indirect ELISA. Results are shown as OD_450_ nm values **(B)** and P/N values **(C)**. **(D)** Isotype determination of mAbs 4D8 and 6F3 using a mouse monoclonal antibody isotyping kit.

The recombinant BCoV N protein purified by affinity chromatography under non-denaturing conditions showed a clear band with a molecular weight of about 66 kDa after SDS-PAGE. This protein was used for subsequent immune experiments (**Figure 1A**). Mice were subcutaneously immunized at multiple sites with 100µg of BCoV N protein per mouse at 2-week intervals. Blood samples were collected after the third immunization, and serum antibody titers were determined using an indirect ELISA. The results were presented as OD_450_ nm values (**Figure 1B**) and P/N values (**Figure 1C**). As shown in **Figure 1**, both mice after immunization produced antibodies against the BCoV N protein, with an antibody titer of 1:51200. Mouse 1 with the highest antibody titer was selected for cell fusion to generate mAbs. Splenocytes of immune mice were fused with SP2/0 cells. After three rounds of subcloning, two hybridoma cell lines that specifically recognized BCoV N protein were obtained, named 4D8 and 6F3 respectively. Antibody isotype identification revealed that both mAbs belong to the IgG1 isotype (**Figure 1D**).

### Characterization of mAbs against BCoV N protein

**Figure 2:**
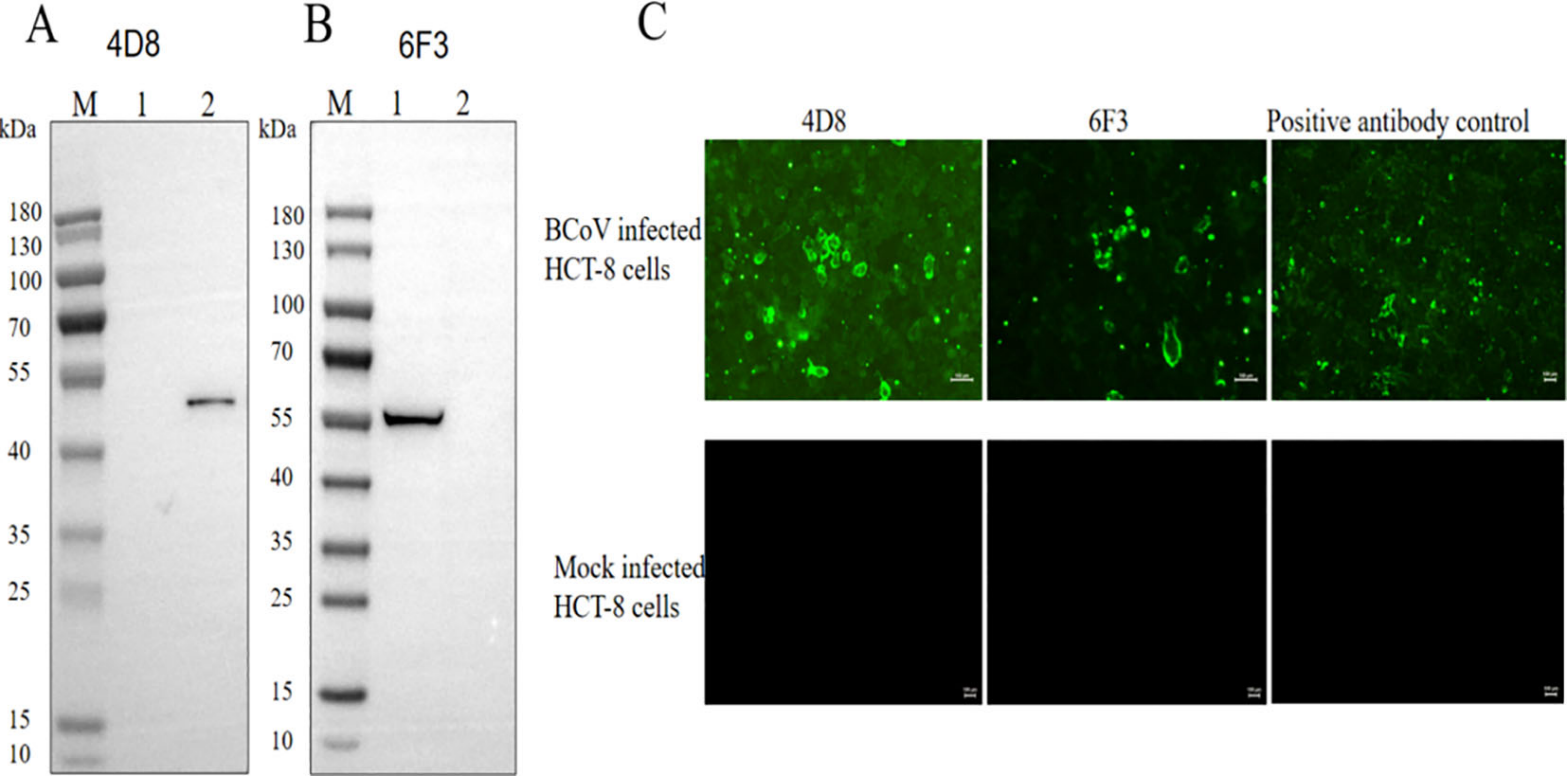
Reactivity identification of mABs. The mABs were identified by Western blot **(A, B)** and IFA **(C)**. **(A)** Lane 1: HCT-8 cell lysate mock-infected with BCoV; Lane 2: HCT-8 cell lysate infected with BCoV; **(B)** Lane 1: lysate of HCT-8 cells infected with BCoV; Lane 2: lysate of mock-infected HCT-8 cells. **(C)** IFA detection of HCT-8 cells infected with BCoV. After inoculation with the BCoV strain, HCT-8 cells were fixed and analyzed using two monoclonal antibodies through IFA. Uninfected cells used as controls.

In order to verify the specificity and reactivity of the prepared monoclonal antibodies, HCT-8 cells infected or uninfected with BCoV were collected. Then these mAbs were used as primary antibodies for subsequent analyses. Western blot results show that both mAbs specifically recognized the BCoV (**Figure 2A**). Consistently, IFA detected specific green fluorescence in HCT-8 cells infected with BCoV, while no fluorescent signal was observed in the control groups (**Figure 2B**).

### Identification and characterization of epitopes recognized by anti-N protein mAbs

**Figure 3:**
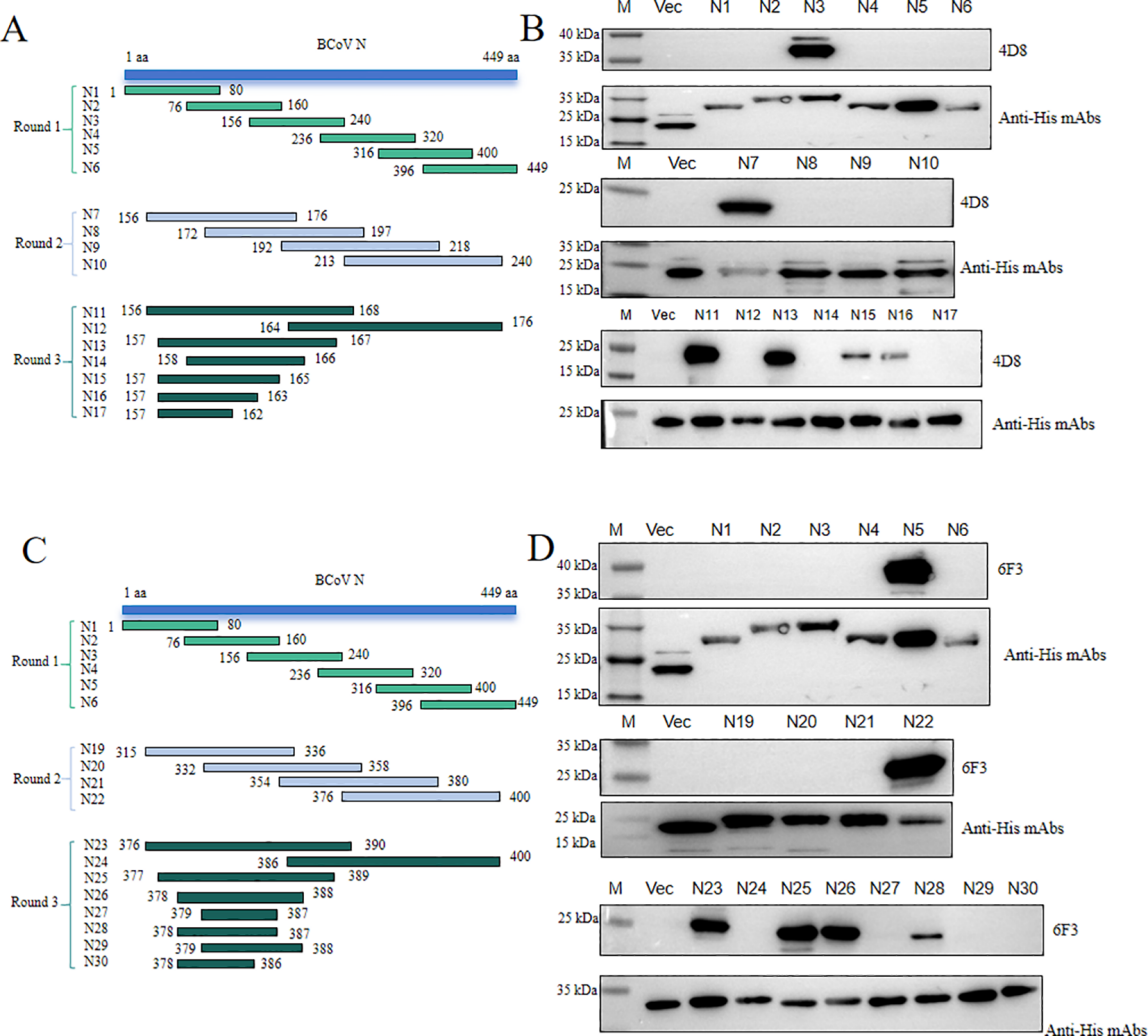
Epitope mapping of mAbs 4D8 and 6F3. **(A, C)** Schematic diagram of BCoV N protein truncated fragments used for epitope positioning. **(B)** Western blot analyzes the reactivity of mAb 4D8 with truncated N protein. mAb 4D8 specifically recognized N3, N7, N11, N13, N15 and N16, indicating that the minimal epitope is ^157^TPADILD^163^. **(D)** Western blot analyzes the reactivity of mAb 6F3 to truncated N protein. mAb 6F3 specifically recognized N5, N22, N23, N25, N26, and N28, indicating that the epitope is ^378^NAYQQQDGMM^387^. M, protein marker; Vec, empty vector control.

To determine the epitopes of anti-N mAbs 4D8 and 6F3, a series of truncated N protein fragments were constructed and expressed. Western blot analysis showed that mAb 4D8 specifically bound to fragment N3 (**Figure 3B**), and mAb 6F3 specifically bound to fragment N5 (**Figure 3D**), indicating that the epitope of 4D8 is located within the 156–240 region, and that of 6F3 within the 315–400 region. In order to obtain accurate epitope, the oligopeptides of N3 and N5 (N7-N17, N19–N30) were further shortened. The results showed that mAb 4D8 bound to the truncated proteins N7, N11, N13, N15 and N16, indicating that ^157^TPADILD^163^ was the exact epitope (**Figure 3B**). Antibody 6F3 reacted with truncated proteins N22, N23, N25, N26 and N28 (**Figure 3D**), and its epitope was determined to be ^378^NAYQQQDGMM^387^.

### Conservation analysis of the identified epitopes

**Figure 4:**
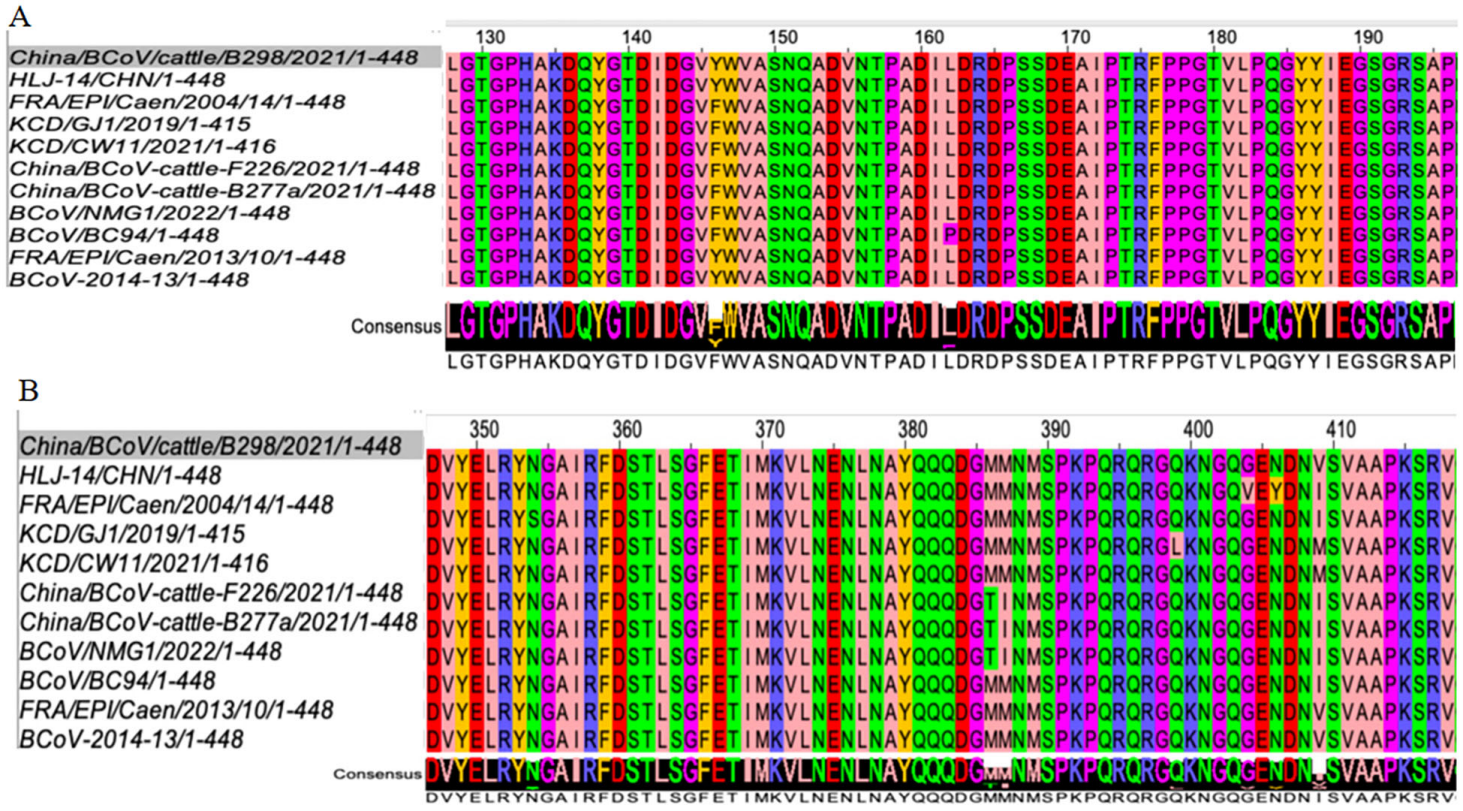
Multiple sequence alignment of the identified antigenic epitopes and flanking regions among BCoV strains. Sequences of the identified epitopes, ^157^TPADILD^163^**(A)**, and ^378^NAYQQQDGMM^387^**(B)**, and different genotypes of BCoV virus strains from GenBank downloaded. Conserved residues are highlighted in black, while variable residues are color-coded according to the physicochemical properties of amino acids.

The conservation of the identified epitopes across different BCoV strains is shown in **Figure 4** and **Table 1**. As shown in **Figure 4**, the regions carrying the epitopes were highly conserved across most of the strains analyzed. The epitope ^157^TPADILD^163^ (**Figure 4A**) showed consistency in all the analyzed strains. For the epitope ^378^NAYQQQDGMM^387^ (**Figure 4B**), occasional amino acid substitution was observed in a few strains in the adjacent region. Specifically, only “62L” in the epitope of one strain is replaced by “62P”. In addition, in some strains, “86M” and “87M” are replaced by “86T” and “87I” respectively. Despite these sporadic mutations, the overall sequence of the epitope region remained highly conserved, indicating that these epitopes may be located in the functionally limited regions of viral proteins.

### Epitope charateristics analysis

**Figure 5:**
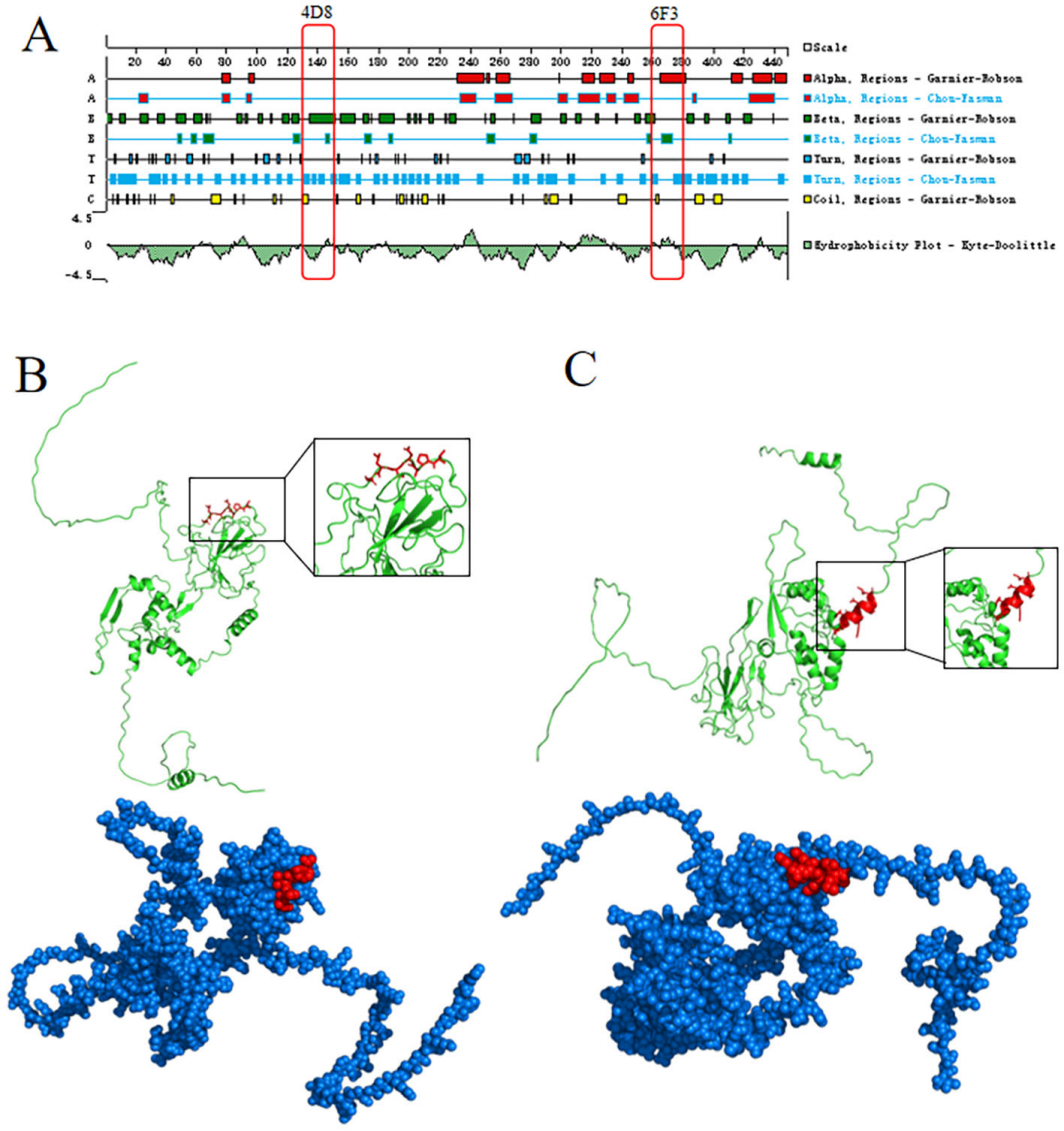
Characterization of antigenic epitopes. **(A)** Prediction of B-cell antigenic epitopes using DNAStar Protean 7.0 software. **(B, C)** Spatial localization of epitopes based on the predicted three-dimensional structure of the BCoV N protein. The tertiary structure of the BCoV N protein was predicted using the SWISS-MODEL homology modeling server, and the epitopes recognized by mAbs 4D8 and 6F3 were mapped onto the predicted model using PyMOL software. **(B)** The epitope ^157^TPADILD^163^ identified by mAb 4D8 is exposed to the protein surface and located in a ring region (highlighted in red). **(C)** The epitope ^378^NAYQQQDGMM^387^ identified by mAb 6F3 is also located in the exposed area of the protein surface (red part). The surface exposure properties of these two epitopes are consistent with their antigen characteristics, supporting their potential use in the diagnostic application and structural research of BCoV N protein.

The analysis showed that the epitope recognized by mAb 4D8, ^157^TPADILD^163^, is mainly located in the region composed of β-sheet and β-turn, suggesting a relatively ordered secondary structure. In contrast, the epitope recognized by mAb 6F3, ^378^NAYQQQDGMM^387^, is mainly located in the region composed of α-helix and random coil, which indicates that its structural configuration is relatively flexible (**Figure 5A**).

The hydrophilicity analysis showed that both epitopes are located in regions with high hydrophilicity scores, indicating good hydrophilicity. In addition, combined with the secondary structure prediction, the two predicted epitopes are exposed to the surface of the protein, which supports their strong antigenicity and accessibility as linear B-cell epitopes. These structural characteristics are consistent with the Western blot and IFA results, which provides a molecular basis for understanding the specific recognition of these antigen epitopes by their respective mAbs.

Structural visualization showed that both epitopes are exposed on the surface of the BCoV N protein. The epitope ^157^TPADILD^163^ recognized by mAb 4D8, is located within a loop region on the protein surface (**Figure 5B**). Similarly, the epitope ^378^NAYQQQDGMM^387^ identified by the monoclonal antibody 6F3 is also located in an exposed area, confirming its accessibility as a linear B cell epitope (**Figure 5C**). The surface exposure characteristics of these two epitopes are consistent with their antigen characteristics and support their potential use in diagnostic applications and BCoV N protein structure research.

## Discussion

Bovine coronavirus (BCoV) is a major enteric and respiratory pathogen of cattle, causing significant economic losses to the global cattle industry (Cho et al., 2001; Hodnik et al., 2020). The nucleocapsid (N) proteinis the most abundant and highly conservative structural protein with strong immunogenicity. It is an ideal target for early diagnosis and vaccine development (Zhou et al., 2025; Xiao et al., 2025; Song et al., 2023). In this study, we successfully generated two novel mAbs against BCoV N protein, 4D8 and 6F3. Western blot and IFA show that the two monoclonal antibodies have high specificity and reactivity to BCoV, with no cross-reactivity against uninfected cells. 4D8 and 6F3 monoclonal antibodies provide valuable tools for the specific detection of BCoV infection and the functional study of N protein.

Accurate identification of B cell epitopes is crucial to understanding the antigen-antibody interaction and the development of epitope-based diagnostic analysis and subunit vaccines (De Leon et al., 2024; Sun et al., 2019). Through systematic truncation of the N protein and Western blot analysis, we mapped the minimal linear epitopes recognized by these mAbs. It is worth noting that the two epitopes ^157^TPADILD^163^ and ^378^NAYQQQDGMM^387^ found in this study are both new epitopes, which have not been reported in previous BCoV N protein studies. A recent study by Zhou et al. (2025) found that there is a conservative B-cell epitope ^380^YQQQDG^385^ is well within our epitope ^378^NAYQQQDGMM^387^. The ^380^YQQQDG^385^ sequence is the core component of the longer epitope we have found. This overlap indicates that the region spanning amino acids 378–387 contains a highly antigenic hotspot. The identification of a longer epitope by our mAb 6F3 may be due to the difference in antibody affinity or the specific configuration of the truncated peptides expressed in the prokaryotic system used for localization, highlighting the complexity of epitope presentation.

It should be noted that the epitope identified by our mAb 6F3 (^378^NAYQQQDGMM^387^) encompasses the recently reported B-cell epitope ^380^YQQQDG^385^ (Zhou et al., 2025). Given the overlapping sequence and the fact that both antibodies were raised against the BCoV N protein, it is reasonable to consider that they recognize essentially the same epitope. The apparent difference in epitope length—with our mAb 6F3 recognizing a longer stretch—likely reflects technical variations between the two studies, such as differences in the design of truncated peptides, the expression systems used for fragment preparation, or the sensitivity of the detection methods employed for epitope mapping. Despite this minor discrepancy, the core recognition sequence remains consistent across both studies, and both antibodies target the same antigenic hotspot within the N protein. Therefore, the epitope ^378^NAYQQQDGMM^387^ can be regarded as a refined definition of the previously identified epitope rather than a distinct novel epitope. In contrast, the epitope ^157^TPADILD^163^ identified by mAb 4D8 represents a genuinely novel linear B-cell epitope that has not been reported in any previous study on BCoV N protein.

The spatial positioning of antigen epitopes on the surface of proteins is a key factor in determining its antigenic and antibody accessibility (Paus and Winter, 2006; Pedroza-Escobar et al., 2023). In this study, we employed SWISS-MODEL for homology modeling and PyMOL for structural visualization of the BCoV N protein epitopes. we found that both ^157^TPADILD^163^ and ^378^NAYQQQDGMM^387^ are exposed on the surface of the BCoV N protein. Epitope 157TPADILD163 is located in a loop region, which is typically flexible and favorable for antibody binding. The epitope ^378^NAYQQQDGMM^387^ forms an α-helical structure. The surface exposure characteristics of these two epitopes confirm the possibility of their potential good targets for diagnostic applications. While SWISS-MODEL is a widely used and reliable tool for template-based protein structure prediction, we acknowledge the emergence of advanced AI-powered platforms such as AlphaFold 3 (Abramson et al., 2024). Unlike SWISS-MODEL, which relies on existing template structures, AlphaFold 3 utilizes a deep learning architecture to predict protein structures with remarkable accuracy, even for regions lacking homologous templates. It also offers enhanced capabilities for predicting protein-protein interactions and post-translational modifications. However, at the time of this study, access to AlphaFold 3 was limited, and SWISS-MODEL provided a sufficiently accurate model for our epitope mapping analysis. Future structural validation of the identified epitopes, particularly their interactions with mAbs 4D8 and 6F3, could benefit from integrating AlphaFold 3-predicted models alongside experimental methods such as X-ray crystallography or cryo-EM. This combined approach may offer deeper insights into the molecular basis of epitope-antibody recognition.

Sequence conservation analysis is crucial for evaluating an antigen epitope in diagnosis or vaccine development. In this study, we use Jalview to compare the amino acid sequences of these antigen epitopes with those of different BCoV reference strains to evaluate their protection. Our results show that the regions carrying epitopes are highly conserved in the vast majority of BCoV strains. It is worth noting that only occasional amino acid replacement was observed in a few strains: residue “162L” was occasionally replaced by “162P”, and the adjacent residues “386M” and “387M” were replaced by “386T” and “387I”, respectively. Despite these minor variations, the core epitope regions maintained a high overall sequence consistency. Regarding the occasional amino acid substitutions observed in a few strains (L162P, M386T, and M387I), it is important to assess their potential impact on antibody binding. Notably, residue 162 lies at the periphery of the epitope ^157^TPADILD^163^, and the substitution from leucine to proline at this position does not alter the core recognition motif (^157^TPADIL^162^). Similarly, residues 386 and 387 are located at the C-terminal flanking region of the epitope ^378^NAYQQQDG^385^, and their substitution is unlikely to abrogate antibody recognition, as the core sequence ^378^NAYQQQDG^385^ remains intact. Therefore, these sporadic mutations are not expected to compromise the diagnostic applicability of the mAbs. In contrast, the adjacent sequence fragments exhibited obvious polymorphism, including additional amino acid substitutions and insertions or deletions.

This high degree of conservation is expected, because the N protein has been proven to be one of the structural proteins with the strongest genetic stability in coronavirus (Zhou et al., 2025). Only occasional replacements were observed in a few virus strains (L162P, M386T and M387I), and these replacements did not destroy the integrity of the overall epitope, which further proved the functional importance of these regions. This finding strongly shows that the mAbs 4D8 and 6F3 and the epitopes they recognize can be used as a general detection tool for BCoV infection worldwide without being affected by virus strain mutations. The high degree of conservation of the ^378^NAYQQQDGMM^387^ epitope overlaps with the previously reported conserved region (Zhou et al., 2025), which further enhances the importance of the domain. In contrast, the epitope ^157^TPADILD^163^ represents a newly identified conserved region of the protein center, expanding the potential targets for BCoV detection. Occasionally observed variations outside these core areas (e.g. positions 162, 386, 387) will not affect the broad-spectrum applicability of these diagnostic targets.

It should be noted that clinical sample validation using ELISA, LFD, or LAT formats was not performed in this study, as the current work focused primarily on mAb generation and epitope mapping. However, IFA and WB have already confirmed that mAbs 4D8 and 6F3 specifically recognize intact BCoV particles. Additionally, the mAbs were produced from ascites without recombinant expression, limiting batch consistency and large-scale production; their performance in complex clinical matrices (e.g., feces or nasal swabs) remains uncharacterized; and validated clinical specimen panels were not available at this stage. These practical constraints will be addressed in future studies following recombinant antibody production.

Beyond the immediate application of these mAbs as detection reagents, we recognize that their long-term utility and scalability for commercial diagnostic development would be greatly enhanced by genetic characterization. Therefore, in our future studies, we plan to determine the full-length nucleotide and amino acid sequences of the variable regions of mAbs 4D8 and 6F3, and subsequently clone their genes into stable expression vectors. This will not only ensure the perpetual and consistent supply of these valuable immunoreagents but also facilitate potential engineering efforts, such as the generation of recombinant antibodies or chimeric formats, thereby broadening their applicability in both research and clinical settings.

In conclusion, this study successfully generated two specific mAbs against the BCoV N protein and identified two novel linear B-cell epitopes, ^157^TPADILD^163^ and ^378^NAYQQQDGMM^387^. These two epitopes are highly conserved among BCoV virus strains and are exposed to the protein surface. These mAbs and their identified epitopes not only provide valuable reagents for the study of the structure and function of BCoV N protein, but also lay a solid foundation for the development of specific, sensitive and widely applicable BCoV diagnostic methods.

## Data Availability

The datasets presented in this study can be found in online repositories. The names of the repository/repositories and accession number(s) can be found in the article. The BCoV N protein sequence used for expression is available in GenBank under accession No. OP866728.1.

## References

[B1] Abramson J. Adler J. Dunger J. Evans R. Green T. Pritzel A. . (2024). Accurate structure prediction of biomolecular interactions with AlphaFold 3. Nature 630, 493–500. doi: 10.1038/s41586-024-07487-w 38718835 PMC11168924

[B2] Bartels C. J. Holzhauer M. Jorritsma R. Swart W. A. Lam T. J. (2010). Prevalence, prediction and risk factors of enteropathogens in normal and non-normal faeces of young Dutch dairy calves. Prev. Vet. Med. 93, 162–179. doi: 10.1016/j.prevetmed.2009.09.020 19819574 PMC7125667

[B3] Burimuah V. Sylverken A. Owusu M. El-Duah P. Yeboah R. Lamptey J. . (2020). Molecular-based cross-species evaluation of bovine coronavirus infection in cattle, sheep and goats in Ghana. BMC Vet. Res. 16, 405. doi: 10.1186/s12917-020-02606-x 33109183 PMC7590242

[B4] Cho K. O. Hasoksuz M. Nielsen P. R. Chang K. O. Lathrop S. Saif L. J. (2001). Cross-protection studies between respiratory and calf diarrhea and winter dysentery coronavirus strains in calves and RT-PCR and nested PCR for their detection. Arch. Virol. 146, 2401–2419. doi: 10.1007/s007050170011 11811688 PMC7087283

[B6] Decaro N. Campolo M. Desario C. Cirone F. D'Abramo M. Lorusso E. . (2008). Respiratory disease associated with bovine coronavirus infection in cattle herds in Southern Italy. J. Vet. Diagn. Invest. 20, 28–32. doi: 10.1177/104063870802000105 18182504

[B5] De Leon A. J. Tjiam M. C. Yu Y. (2024). B cell epitope mapping: The journey to better vaccines and therapeutic antibodies. Biochim. Biophys. Acta Gen. Subj. 1868, 130674. doi: 10.1016/j.bbagen.2024.130674 39079649

[B7] Gagea M. I. Bateman K. G. van Dreumel T. McEwen B. J. Carman S. Archambault M. . (2006). Diseases and pathogens associated with mortality in Ontario beef feedlots. J. Vet. Diagn. Invest. 18, 18–28. doi: 10.1177/104063870601800104 16566254

[B8] He R. Leeson A. Ballantine M. Andonov A. Baker L. Dobie F. . (2004). Characterization of protein-protein interactions between the nucleocapsid protein and membrane protein of the SARS coronavirus. Virus Res. 105, 121–125. doi: 10.1016/j.virusres.2004.05.002 15351485 PMC7127797

[B9] Hodnik J. J. Ježek J. Starič J. (2020). Coronaviruses in cattle. Trop. Anim. Health Prod. 52, 2809–2816. doi: 10.1007/s11250-020-02354-y 32681447 PMC7367658

[B10] Hurst K. R. Koetzner C. A. Masters P. S. (2013). Characterization of a critical interaction between the coronavirus nucleocapsid protein and nonstructural protein 3 of the viral replicase-transcriptase complex. J. Virol. 87, 9159–9172. doi: 10.1128/JVI.01275-13 23760243 PMC3754073

[B11] Kanno T. Hatama S. Ishihara R. Uchida I. (2007). Molecular analysis of the S glycoprotein gene of bovine coronaviruses isolated in Japan from 1999 to 2006. J. Gen. Virol. 88, 1218–1224. doi: 10.1099/vir.0.82656-0 17374765

[B12] Keha A. Xue L. Yan S. Yue H. Tang C. (2019). Prevalence of a novel bovine coronavirus strain with a recombinant hemagglutinin/esterase gene in dairy calves in China. Transbound Emerg. Dis. 66, 1971–1981. doi: 10.1111/tbed.13228 31077561 PMC7168545

[B13] Kopecky-Bromberg S. A. Martínez-Sobrido L. Frieman M. Baric R. A. Palese P. (2007). Severe acute respiratory syndrome coronavirus open reading frame (ORF) 3b, ORF 6, and nucleocapsid proteins function as interferon antagonists. J. Virol. 81, 548–557. doi: 10.1128/JVI.01782-06 17108024 PMC1797484

[B14] Lai M. M. Cavanagh D. (1997). The molecular biology of coronaviruses. Adv. Virus Res. 48, 1–100. doi: 10.1016/S0065-3527(08)60286-9 9233431 PMC7130985

[B15] Lojkić I. Krešić N. Šimić I. Bedeković T. (2015). Detection and molecular characterisation of bovine corona and toroviruses from Croatian cattle. BMC Vet. Res. 11, 202. doi: 10.1186/s12917-015-0511-9 26268320 PMC4535285

[B16] Narayanan K. Kim K. H. Makino S. (2003). Characterization of N protein self-association in coronavirus ribonucleoprotein complexes. Virus Res. 98, 131–140. doi: 10.1016/j.virusres.2003.08.021 14659560 PMC7125804

[B17] Park S. Jeong C. Yoon S. Choy H. Saif L. J. Park S. . (2006). Detection and characterization of bovine coronaviruses in fecal specimens of adult cattle with diarrhea during the warmer seasons. J. Clin. Microbiol. 44, 3178–3188. doi: 10.1128/JCM.02667-05 16954245 PMC1594715

[B18] Paus D. Winter G. (2006). Mapping epitopes and antigenicity by site-directed masking. Proc. Natl. Acad. Sci. U.S.A. 103, 9172–9177. doi: 10.1073/pnas.0600263103 16754878 PMC1482585

[B19] Pedroza-Escobar D. Castillo-Maldonado I. González-Cortés T. Delgadillo-Guzmán D. Ruíz-Flores P. Cruz J. H. S. . (2023). Molecular bases of protein antigenicity and determinants of immunogenicity, anergy, and mitogenicity. Protein Pept. Lett. 30, 719–733. doi: 10.2174/0929866530666230907093339 37691216

[B20] Saif L. J. (2010). Bovine respiratory coronavirus. Vet. Clin. North. Am. Food. Anim. Pract. 26, 349–364. doi: 10.1016/j.cvfa.2010.04.005 20619189 PMC4094360

[B21] Saif L. J. Jung K. (2020). Comparative pathogenesis of bovine and porcine respiratory coronaviruses in the animal host species and SARS-CoV-2 in humans. J. Clin. Microbiol. 58, e01355-20. doi: 10.1128/JCM.01355-20 32522830 PMC7383540

[B22] Song W. Fang Z. Ma F. Li J. Huang Z. Zhang Y. . (2023). The role of SARS-CoV-2 N protein in diagnosis and vaccination in the context of emerging variants: present status and prospects. Front. Microbiol. 14, 1217567. doi: 10.3389/fmicb.2023.1217567 37675423 PMC10478715

[B23] Sun P. Guo S. Sun J. Tan L. Lu C. Ma Z. (2019). Advances in in-silico B-cell epitope prediction. Curr. Top. Med. Chem. 19, 105–115. doi: 10.2174/1568026619666181130111827 30499399

[B24] Surjit M. Liu B. Chow V. T. Lal S. K. (2006). The nucleocapsid protein of severe acute respiratory syndrome-coronavirus inhibits the activity of cyclin-cyclin-dependent kinase complex and blocks S phase progression in mammalian cells. J. Biol. Chem. 281, 10669–10681. doi: 10.1074/jbc.M509233200 16431923 PMC7995956

[B25] van den Hurk S. Regmi G. Naikare H. K. Velayudhan B. T. (2024). Advances in laboratory diagnosis of coronavirus infections in cattle. Pathogens 13, 524. doi: 10.3390/pathogens13070524 39057751 PMC11279749

[B26] Vlasova A. N. Saif L. J. (2021). Bovine coronavirus and the associated diseases. Front. Vet. Sci. 8, 643220. doi: 10.3389/fvets.2021.643220 33869323 PMC8044316

[B28] Waterhouse A. Bertoni M. Bienert S. Studer G. Tauriello G. Gumienny R. . (2018). SWISS-MODEL: homology modelling of protein structures and complexes. Nucleic Acids Res. 46, W296–W303. doi: 10.1093/nar/gky427 29788355 PMC6030848

[B27] Waterhouse A. M. Procter J. B. Martin D. M. Clamp M. Barton G. J. (2009). Jalview version 2—a multiple sequence alignment editor and analysis workbench. Bioinformatics 25, 1189–1191. doi: 10.1093/bioinformatics/btp033 19151095 PMC2672624

[B29] Xiao Y. Song Z. Zhou L. Lu W. Fang W. Xu J. . (2025). Coronavirus nucleocapsid proteins: a multifaceted modulator in the innate immune evasion. Front. Microbiol. 16, 1658339. doi: 10.3389/fmicb.2025.1658339 41445958 PMC12723872

[B30] Zhou Y. Zhang J. Wu H. Zhao S. Ren Y. Chen Q. . (2025). First linear B-cell epitope identified on the nucleocapsid protein of bovine coronavirus. Virology 610, 110581. doi: 10.1016/j.virol.2025.110581 40483734

[B31] Zhu Q. Li B. Sun D. (2022). Advances in bovine coronavirus epidemiology. Viruses 14, 1109. doi: 10.3390/v14051109 35632850 PMC9147158

